# Twenty-two years of dengue outbreaks in Bangladesh: epidemiology, clinical spectrum, serotypes, and future disease risks

**DOI:** 10.1186/s41182-023-00528-6

**Published:** 2023-07-11

**Authors:** Mohammad Sorowar Hossain, Abdullah Al Noman, SM Abdullah Al Mamun, Abdullah Al Mosabbir

**Affiliations:** 1 Department of Emerging and Neglected Diseases, Biomedical Research Foundation, Dhaka, Bangladesh; 2 Department of Environmental Science and Management, Independent University, Bangladesh, Dhaka, Bangladesh; 3 Department of Hematology & BMT Unit, Dhaka Medical College Hospital, Dhaka, Bangladesh

## Abstract

Dengue is the most rapidly spreading mosquito-borne disease and has become a major public health threat, particularly for tropical and subtropical countries including Bangladesh. This comprehensive review aims to summarize the overall scenario of dengue, including disease burden, clinical spectrum, seroprevalence, circulating serotypes/genotypes, and spatial distribution since the first recorded outbreak in Bangladesh. Since the first recorded outbreak in 2000, dengue epidemiology has shown the typical epidemic pattern with more frequent and bigger outbreaks and gradual geographic expansion to non-endemic regions in Bangladesh. For instance, highly confined Rohingya refugee camps that provide shelters to nearly 1.2 million forcibly displaced vulnerable Myanmar nationals in Cox’s Bazar district confronted a massive outbreak in 2022. Recent major outbreaks are found to be associated with the emergence of serotype DENV-3, which was undetected for a long time. Consequently, changes in serotypes might be attributed to increased severity in clinical manifestation in recent years. The existing weak surveillance and risk management systems are inadequate to deal with impending dengue risks. The healthcare system, particularly at the district level, is not prepared to manage impending large-scale dengue outbreaks in Bangladesh. Our findings would contribute to the development of strategies for dengue control and management in Bangladesh as well as other similar settings elsewhere in the world.

**Supplementary Information:**

The online version contains supplementary material available at 10.1186/s41182-023-00528-6.

## Background

Dengue, the fastest-spreading mosquito-borne infectious disease, has emerged as a global public health problem [1]. The World Health Organization considers dengue among the top ten global health threats [2]. This is particularly concerning as no specific treatment or vaccine against dengue is not widely available (a dengue vaccine developed by Sanofi Pasteur, has been licensed in 24 countries and introduced into public immunization programs in the Philippines and Brazil) [3]. Furthermore, the dengue virus’s genetic heterogeneity (serotype and genotype) adds another dimension to the public health challenge due to the increased risk of disease severity (secondary and tertiary infection). Dengue has spread over 125 countries, with 400 million annual infections and 40,000 deaths [1]. The endemic regions of tropical and subtropical countries (South East Asia and South Asia) account for 70% of the dengue burden [1].

Under changing climate due to global warming, dengue is expected to spread more into regions with immunologically naive populations in sub-Saharan Africa, parts of Europe and the northern USA, and lowland areas of the Western Pacific and Eastern Mediterranean regions [4]. The climate suitability of dengue transmission is also predicted to increase by four additional months, and about 1.4 billion additional people (altogether 4.7 billion) will be at risk [5].

Low- and middle-income countries (LIMCs) with higher population density, poor healthcare systems, rapid unplanned urbanization, and global warming-induced changing climatic factors are particularly vulnerable to dengue [6]. More than half of the estimated dengue infections (31,245,000/56,879,000) occurred between 1990 and 2019 in South Asia [7, 8]. Besides, dengue claimed the lives of 20,837 individuals during this period which is a 140% increase from 1990.

Bangladesh, a South Asian country with over 165 million population, has been a dengue-endemic country since the first recorded outbreak in 2000. Due to multiple risk factors, Bangladesh has been experiencing successive major dengue outbreaks in recent years. Given the fact that if proper preventative strategies are not implemented, poor healthcare infrastructure, inadequate outbreak preparedness, and the lack of community-level awareness of dengue infection may lead to public health disasters.

This comprehensive review aims to depict an overall scenario of dengue (disease burden, spatial distribution, clinical manifestations, seroprevalence, circulating serotypes, and genotypes) after the first outbreak in Bangladesh.

## Methodology

### Search strategy

**Fig. 1:**
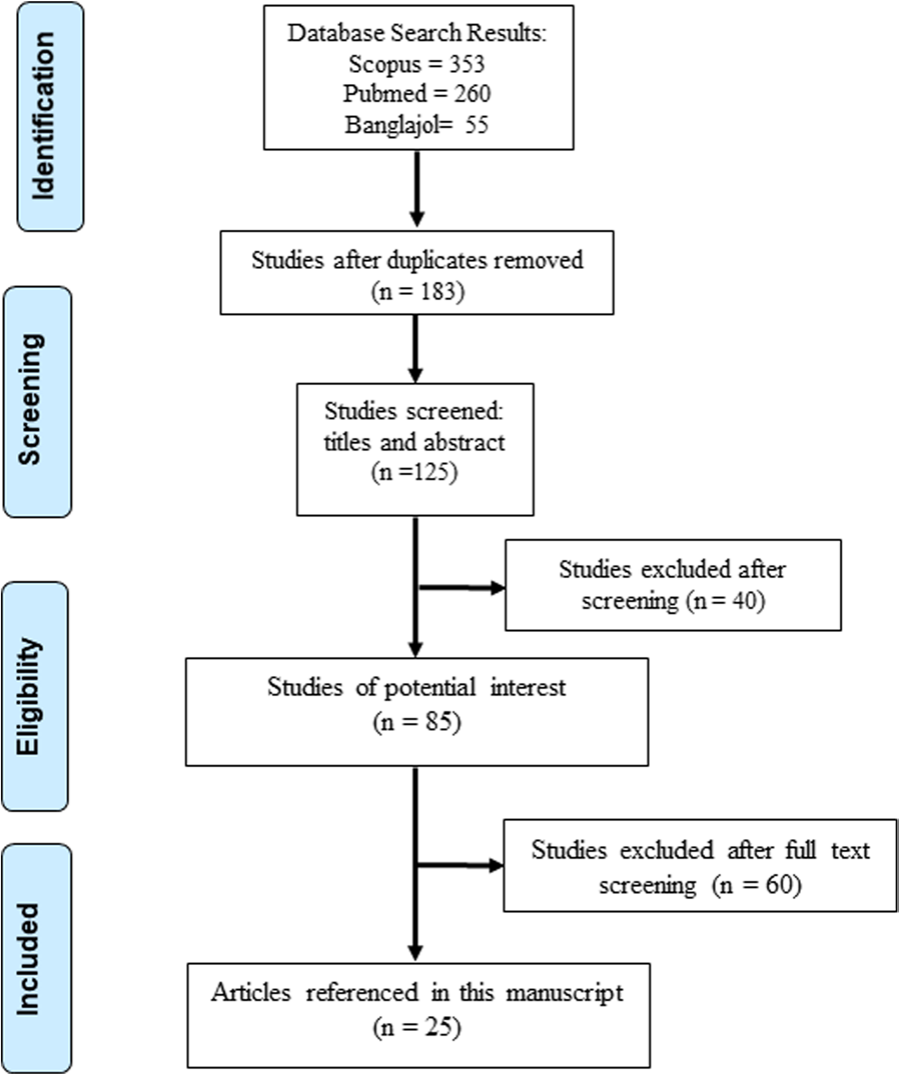
Systematic search for journal articles describing dengue in Bangladesh

A narrative literature review was conducted on the current status of epidemiology, clinical spectrum, serotypes, and environmental factors in Bangladesh. However, we did not register any protocol for this review. For this purpose, Scopus and PubMed were searched using the keyword “Dengue AND Bangladesh”. The date of publication of articles was limited to 1 January 2000 to 30 December 2022. Bangladeshi articles were searched in the BanglaJol database (an exclusive database of Bangladeshi Journal) with a combination of keywords “dengue”, “Bangladesh”, “epidemiology”, “outbreak”, “temperature”, “clinical features”, “global warming”, etc. Figure 1 depicts the systematic search of published articles and Additional file 1**:** File 1 presents the search strategy for PubMed as performed in January 2023.

### Eligibility criteria

In this study, specific criteria were used to select relevant articles for analysis. The inclusion criteria were: (i) articles describing the epidemiology, clinical manifestations, serotype/genotype, environmental factors and knowledge and awareness regarding dengue in Bangladesh and (ii) articles published in English. The exclusion criteria were: (i) non-peer-reviewed articles (such as editorials and comments) and (ii) articles published in other languages.

### Selection of studies and data extraction

The final search results were inserted into Excel and duplicates were removed. Two reviewers (MSH and AAN) screened the title and abstract. Full texts of the primarily selected articles were also screened thoroughly. Any discrepancies found during the selection of studies were resolved through discussion and consensus of the two reviewers which were then evaluated by a third reviewer (AAM).

### Quality assessment

The quality of the studies was assessed using the Scale for the Assessment of Narrative Review Articles (SANRA) [9]. The scale consists of six items and is rated in integers from 0 (low standard) to 2 (high standard). The maximal sum score is 12. The definition, explanation and example for each item are attached to the scale to improve clarity and assist users in completing the scale. Similar to screening, quality assessment was also performed by two independent reviewers (MSH and AAN) and any discrepancies found were resolved through discussion and consensus of the two reviewers.

## Results

### Literature search strategy and quality assessment

Initially, 183 articles were identified via initial searching after removing duplicates and 125 were selected after screening title and abstract. We excluded 40 articles after reading the full-text, and finally, 25 articles were included and discussed in this review. Additional file 1: File 2 listed the 131 selected studies after screening title and abstract. The mean score of the SANRA scale for this article is 10.5, albeit there are no established cutoffs for the quality. The scoring of a reviewer is available in the Additional file 1**:** File 3.

Moreover, in this work, for generating spatial Maps, publicly available data on dengue cases were obtained from the Directorate GGeneral of Health Services, Bangladesh [10] and secondary data on dengue cases from outside of Dhaka without travel history (this study was ethically approved by Biomedical Research Foundation, Bangladesh) [11] and mosquito-related data (this study was ethically approved by icddr,b Bangladesh) [12] were collected from previous studies.

### Major outbreaks (2000–2022) in Bangladesh: a brief history

Dengue incidence was sporadically reported between 1964 and 1999 in Bangladesh until the first major outbreak occurred in 2000 when 5551 hospitalized [11, 12] cases and 93 deaths were recorded [13]. Since then, dengue has become an endemic disease causing thousands of infections and affecting the quality of life of the population. Up to 2021, the capital, Dhaka city was the epicenter of all significant outbreaks [14]. Based on available hospital-based surveillance data, seven outbreaks with more than 5,000 hospitalized cases were recorded over the years (2000–2022) in Dhaka city. After a hiatus, dengue started to increase again in 2016, and the largest dengue outbreak occurred in 2019 when over 100,000 individuals were hospitalized and claimed 179 lives in Dhaka city [15]. This outbreak was followed by another large outbreak in 2021 (28,429 cases and 85 deaths), of which the true magnitude is believed to be masked due to the COVID-19 pandemic (delta onslaught) [14]. The second largest outbreak occurred (61,732 cases, 281 deaths) in 2022 [10]. Simultaneously, another large outbreak was documented in Rohingya refugee camps at Ukhiya, Cox’s Bazar (13,886 cases, 27 deaths) [16] in 2022. The higher case fatality rate (1.67%) in the 2000 outbreak was attributed to the emergence of dengue virus (DENV) in a large naïve (immunologically unchallenged) population for the first time and the unpreparedness of the healthcare system against infectious diseases. In contrast, a lower case fatality rate (< 0.5%) was observed in recent outbreaks **(**Table 1), which is perhaps because of better clinical management as compared to those initial outbreaks [10].

**Table 1:** Summary of dengue outbreak in Bangladesh (2000–2022)

Year	Hospitalized cases	Number of deaths	Case fatality rate (CFR)
2000	5551	93	1.67
2001	2430	44	1.81
2002	6232	58	0.93
2003	486	10	2.05
2004	3434	14	0.40
2005	1048	4	0.38
2006	2200	11	0.50
2007	466	0	0
2008	1153	0	0
2009	474	0	0
2010	409	0	0
2011	1359	6	0.44
2012	671	1	0.15
2013	1749	2	0.11
2014	375	0	0
2015	3162	6	0.19
2016	6060	14	0.23
2017	2769	8	0.29
2018	10,148	26	0.26
2019	101,354	179	0.17
2020	1193	3	0.25
2021	28,429	105	0.37
2022	61,732	281	0.45

### Surveillance system and under-reporting of dengue in Bangladesh

The Directorate General of Health Services (DGHS), Bangladesh, officially initiated the hospital-based dengue surveillance system during the first major outbreak in 2000, where all suspected, probable, and confirmed cases were considered [17]. However, the case definition was updated in 2010 to include only serologically confirmed cases in the surveillance system [17]. Consequently, the current tracking system inherently underestimates the dengue burden in Bangladesh, because many asymptomatic and mild dengue cases are missed out. In addition, this surveillance system was mostly Dhaka city based until the 2019 outbreak when DGHS systematically started collecting district-wise (64 administrative districts) dengue cases. In Dhaka city, only 50 hospital hospitals (17 public and 33 private) out of several hundred hospitals/clinics are assigned to report dengue cases to the current surveillance system [10]. Moreover, several barriers could contribute to the profoundly under-ascertainment and under-reporting of dengue in Bangladesh. These include a lack of healthcare infrastructure at the district level, limited financial resources, and cultural beliefs that may discourage seeking medical care [18]. These barriers can be particularly challenging for women, who may face additional obstacles such as a lack of mobility, social norms that limit their ability to travel or interact with male healthcare providers, and a lack of education or awareness about dengue fever [19, 20].

### Seroprevalence

Serological studies, primarily conducted in Dhaka city, indicate that dengue has been rising over the years. Even though most of the studies cannot be generalizable due to reporting of a few hospitals and small sample size, these may provide useful information regarding age, sex, and other sociodemographic distribution. As of today, only a nationally representative seroprevalence study conducted from 2014 to 2015 revealed that 24% of tested individuals (*n* = 5866) had a previous history of dengue across the country, and over 80% were seropositive among the study population in two major cities (Dhaka and Chittagong) [12]. This seroprevalence study estimated that 40 million individuals had been infected in Bangladesh, with 2.4 million annual dengue cases [12].

### Clinical manifestations of dengue in the last two decades

#### Sex distribution

Gender distribution showed a clear male predominance in all the dengue outbreaks reported in Bangladesh. The proportion of male cases was almost double compared to females in all studies (Table 2). Male-to-female ratio was as high as 2.7 [21]. In adolescents and adults, significant male excess was also noted in six other culturally and economically diverse Asian countries. However, the difference was not significant in pediatric groups [22]. A study among children in Bangladesh also reported a similar result [23]. While male predominance was also reported in most studies from India, few studies showed variable distribution [24]. This is in contrast to findings in South America, where female cases were equal to or greater than males [25]. The clinical significance of such gender differences is not clear. A population-based serosurveillance study conducted in Mexico (2012) found no significant sex difference in seroprevalence [26]. Therefore, this difference could reflect case selection bias as most studies were hospital-based, with male patients prevalent in developing countries, such as Bangladesh. Another possibility could be the cultural aspect (dress code), where females are generally covered in South Asia.

**Table 2:** Describes the detailed demographic distribution and clinical features among dengue patients in major outbreaks

	2000 Outbreak (First outbreak) [27]	2002 Outbreak [21]	2006–2008 Outbreak (Pediatric cases) [38]	2008 Outbrak (DHF cases) [65]	2016 Outbreak [30]	2018 Outbreak [28]	2019 Outbreak [29]	2019 Outbreak (Pediatric cases) [23]	2019 Outbreak (Non endemic Zone) [39]
Sample size (*n*)	176	100	54	55	40	350	553	190	319
Mean age (years)	–	29	6.5	–	–	25	27	8.8	33
Age range (years)	All age	10–70	Less than 16	13 and above	All age	All age	3–85	Less than 15	18 and above
Gender distribution (%)									
Male	–	73	50	84	62	68	63	55	70
Female	–	27	50	16	38	32	37	45	30
Common symptoms (%)									
Fever	100	100	63^a^	100	100	100	100	100	92
Headache	91	96	31	32	25	61	62	68	73
Arthralgia/ Joint pain	85	91	18	09	73	23	04	49	–
Rash	55	28	76		12	6	04	28	16
Retro-orbital pain	–	–	15	–	50	20	39	34	47
Myalgia/body ache	–	–	46	45	12	44	–	37	71
Sore throat/Mouth sore	–	–	–	–	–	–	01	28	–
Lethargy	–	–	–	–	30	–	–	78	–
GI symptoms (%)									
Vomiting	64	93	57	40	27		69	80	34
Abdominal pain	–	83	59	02	30	32	86	65	30
Diarrhoea	–	–	09	04	25	05	26	43	43
Constipation	–	–	–	–	–	–	–	73	04
Anorexia	–	–	–	05	–	–	–	79	–
Bleeding symptoms (%)									
Malena	20	50	15	11	07	–	05	06	12
Gum bleeding	11	41	13	18			03		08
Sub-conjunctival haemorrhage	–	17	33	16	20	–	–	–	09
Epistaxis	–	02	–	02	–	–	01	06	02
Signs of plasma leakage (%)									
Hypotension	–	–	11	71	–	–	25	–	–
Oedema		07	–	–	–	–	0.3	09	01
Pleural effusion	13		28	–	–	–	02	–	02
Ascites	09	–	15	–	–	–	02	–	01
Oliguria/Anuria	–	–	–	–	–	–	2	21	
Atypical manifestations (%)									
Hepatomegaly	07	–	31	–	–	–	01	–	07
Splenomegaly	–	–	03	–	–	–	0.5	–	–
Eye redness	–	–	–	–	–	–	12	–	–
Respiratory distress	–	–	–	–	–	–	04	13	02
Cough	–	–	04	–	–	–	05		03
Convulsion	–	–	–	–	–	–	0.2		–
Jaundice	–	01	–	–	12	–	01	–	–
Excessive sweating	–	–	–	–	–	–	–	40	
Confusion	–	–	–	–	–	–	–	21	–
Blurring of vision	–	–	–	–	–	–	–	12	–
Palpitation	–	–	–	–	–	–	–	08	–
Impaired consciousness	–	–	–	–	–	–	–	20	–
Laboratory abnormalities (%)									
Low Haemoglobin	–	–	–	–	–	–	11	38	–
Thrombocytopenia	57	86	69	100	–	–	66	87	30
Leukopenia	–	–	09	–	–	–	29	40	63
Elevated haematocri^t^	–	–	–	–	–	–	–	13	23
Raised Bilirubin	–	–	–	–	–	–	13	–	–
Raised AST	–	–	–	–	–	–	67	–	05
Raised ALT	–	–	41	–	–	–	50	–	12

Data are presented as *n* (%) unless stated otherwise

a Fever > 5 days

b > 20% from baseline

*ALT* alanine transaminase, *AST* aspartate transaminase

#### Age distribution

Young adults were predominantly affected by dengue in Bangladesh. During the first epidemic in 2000, more than 80% of cases were adults (> 18 years of age); the peak number of cases occurred between 18 and 33 years of age [27]. Likewise, the majority (62%) of the confirmed case belonged to the 16–30 age group, with a mean age of 29 years in the 2002 outbreak [21]. Older adolescents and young adults also comprised the majority of the cases in 2016 (21–40; 55%), 2018 (15–29; 65%), and 2019 (21–40; 50%) outbreaks [28–30]. A similar result was reported in Sri Lanka in 2018 [31] and Ethiopia in 2017 [32]. While studies conducted in different parts of India also noted a predominance of young adults [24], the first major dengue outbreak in Delhi in 1996 mainly affected the 5–12-year-old age group [33]. Similarly, surveillance data in Puerto Rico reported the highest incidence between 10 and 19 years during the 1994 and 1995 outbreaks [34]. This suggests that dengue is experiencing a demographic shift to older ages over the last decade.

Among pediatric cases below 15 years, dengue affected mostly older children in Bangladesh. A hospital-based study conducted during the 2019 outbreak found that the majority (46.1%) of the children belonged to the 10–14 age group with a mean age of 8.8 years [23]. A similar observation was also noted in other Asian countries [35]. The disproportionately high exposure to infected mosquitoes among children > 6 years could explain this as children begin attending elementary school and spend more time in crowded places at that age [36].

Age is also associated with the severity of the disease. Older age in adults and younger age in children were found to be associated with the progression of severe disease in a systematic review and meta-analysis [37]. The authors implicated multiple comorbidities in older adults and increased vascular filtration capacity in young children to be the cause. Likewise, a multicentric hospital-based study in Bangladesh reported that the mean age of severe dengue (7.4 years) was significantly lower than non-severe dengue (9.4 years) [23]. Unfortunately, a comparative study in adults is absent.

#### Clinical spectrum and severity of dengue

During the first outbreak in 2000, the predominant symptoms were fever (100%), headache (91%), and joint pain (85%), which are typical of dengue fever [27] (Table 2). About half of the patients had bleeding manifestations, mainly melena and bleeding gums. Less than 1% of patients presented with dengue shock syndrome (DSS). A similar pattern of the presentation was also noted in the 2002 outbreak [21], except for a very high incidence of gastrointestinal (GI) manifestations. More than 80% of cases reported GI symptoms, especially abdominal pain and vomiting. GI symptom was also reported as the predominant presentation among children during outbreaks between 2006 and 2008 [38].

Bangladesh experienced the largest dengue outbreak in 2019. Apart from fever, the most predominant presentation was again GI symptoms, especially abdominal pain (84.6%) and vomiting (69.2%) [29]. Joint pain/arthralgia was reported by only 4.5% of respondents, while this was a common presentation in previous outbreaks. Abdominal pain, vomiting, and diarrhea were also reported as common manifestations of dengue infection in the non-endemic zone of Bangladesh [39]. The incidence of bleeding manifestations was low (about 5%) in the 2019 outbreak. The most common bleeding manifestations were melena (5%) and gum bleeding (3%) [40]. Notably, DSS was reported in up to 10% of cases compared to only 0.6% in the first outbreak. Besides, hypotension, a feature of plasma leakage and impending shock, was recorded in about one-fourth of the cases in the 2019 outbreak [29]. Other signs of plasma leakage, including edema, and ascites, pleural effusion, were also noted. On the other hand, signs of shock were recorded only in 11% of cases in a study conducted among pediatric patients between 2006 and 2008 [38]. Therefore, it is discernible that dengue is possibly going through an epidemiological shift towards more severe disease (i.e., DSS rather than DHF) in Bangladesh. The resurgence of the DENV-3 serotype could be a possible driver for such change [41]. In addition, it is important to note that the World Health Organization (WHO) updated the case definition of severe dengue in 2009. Compared to the 1997 classification system, the 2009 classification is more sensitive in detecting severe dengue cases, especially cases with DSS [42, 43]. The revised case definition could partly contribute to the higher prevalence of severe cases observed in recent outbreaks. Nonetheless, this is alarming for Bangladesh as future outbreaks could be devastating and claim more lives.

Clinical features in children were different from that of adults. A hospital-based study among the pediatric population in the 2019 outbreak found that besides fever, GI symptoms (vomiting, anorexia, constipation, etc.) were the most common presentation, followed by lethargy and headache [23]. In addition, this same study reported that constipation (72.7%) and mouth sores (28.3%) were prevalent GI symptoms among children, which were previously less reported in dengue infection. A comparison between adults and children showed that the incidence of GI symptoms, headache, retro-orbital pain, and melena was significantly higher among children in the 2019 outbreak, while hypotension incidence was significantly lower than among adults [40].

### Circulating serotypes and genotypes in Bangladesh

The information on the evolution and diversity of the dengue virus is sparse in Bangladesh. Most studies described the genotypes of dengue based on small sample size (Table 3). In early outbreaks (2000–2002), DENV-3 was the prevalent serotype, albeit all four serotypes were circulating. Afterward, there were no data available for serotypes until 2012. Later, Muraduzzaman et al. revealed that DENV-1 and DENV-2 were the prevalent circulating serotypes between 2013 and 2016 [44]. However, DENV-3 emerged after a hiatus in 2017 with the prevalent serotype of DENV-2. Since the largest outbreak in 2019, DENV-3 has been the most prevalent circulating serotype (Table 3). Not much information is available on the genotypic variability of DENV. Three genotypes (I, II, II) of DEN-3 and the cosmopolitan genotype of DEN-2 are currently circulating in Bangladesh. Arguably, the emergence of the DENV-4 serotype, which has been missing for more than 20 years, could pose a significant public health threat to Bangladesh because of secondary infection.

**Table 3:** Circulating serotypes/genotypes of dengue virus in Bangladesh

Year	Sample size (*n*)	DEN1 n (%)	DEN2 *n* (%)	DEN3 *n* (%)	DEN4 *n* (%)	Genotype	References
2000	44	6 (16.6%)	3 (6.8%)	31 (70.5%)	4 (9.1%)		[66]
2002	8	0	0	8 (100%)	0	DEN3-II	[21]
2013	28	4 (14.3%)	24 (85.7%)	0	0		[44]
2014	30	12 (40%)	18 (60%)	0	0		[44]
2015	42	21 (50%)	21 (50%)	0	0		[44]
2016	41	10 (24.4%)	31 (75.6%)	0	0		[44]
2017	151	9 (5.9%)	62 (41.1%)	47 (31.1%)	0		[41]
	161	7 (4.3%)	147 (91.3%)	7 (4.3%)	0		[67]
2018	127	33 (26%)	52 (41%)	42 (33%)	0		[67]
	24	0	13 (54.2%)	11 (47.8%)	0	Cosmopolitant, DENV3-I	[68]
2019	86	7 (8.2%)	0	79 (91.8%)	0		[67]
	41	0	1	40 (99%)	0	DENV2-I, II, II	[69]
2020	1	0	0	1 (100%)	0		[67]
2021	178	0	0	178 (100%)	0		[67]

### Seasonality of dengue outbreak in changing climate

**Fig. 2:**
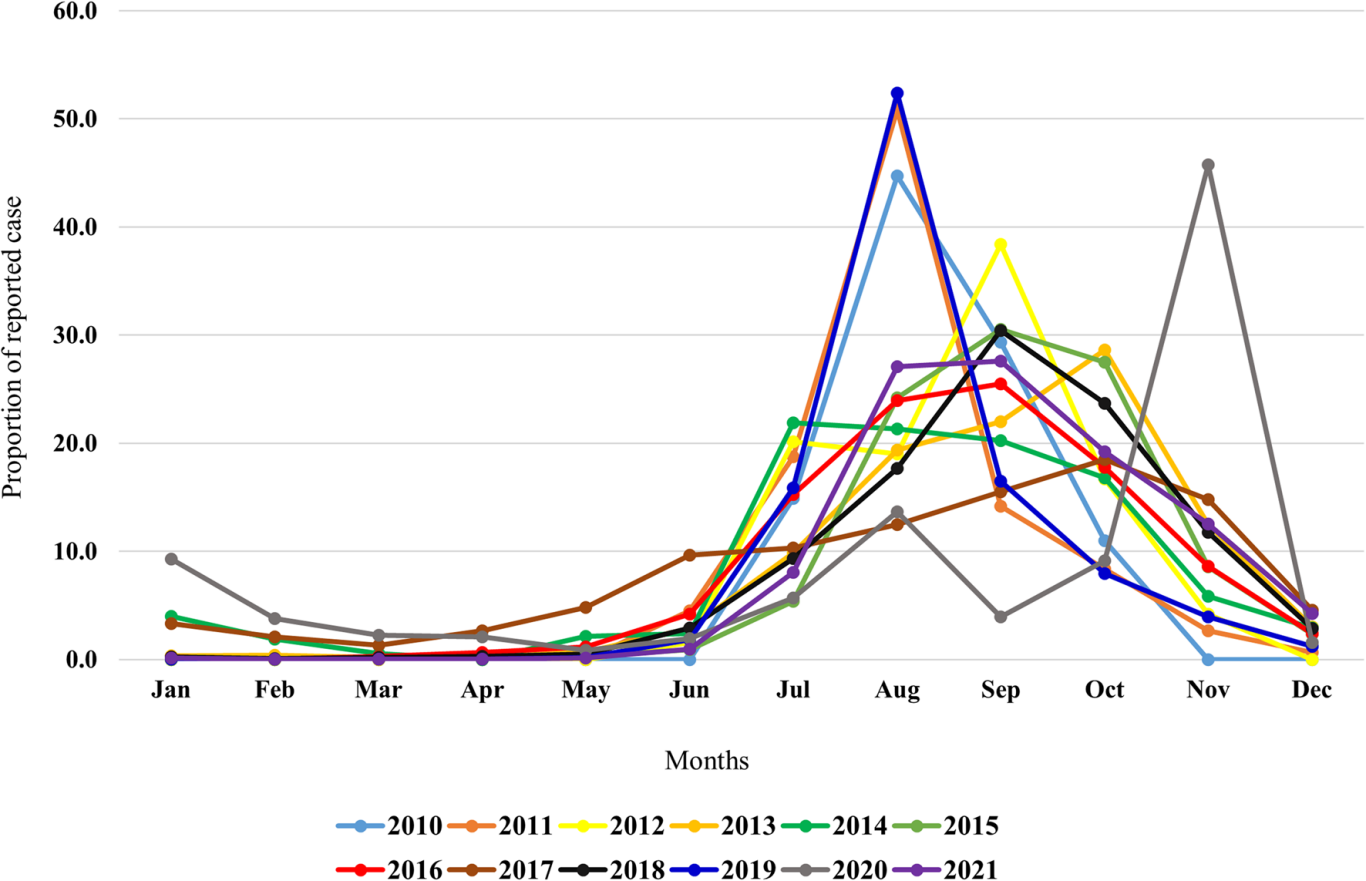
Seasonality of dengue outbreaks in Bangladesh between 2010 and 2021. Publicly available data were obtained from the Directorate general of health services, Bangladesh

Dengue cases are mostly recorded during the monsoon (June–September) in Bangladesh and post-monsoon (October–November) seasons, with a peak in September in terms of higher incidence (Fig. 2). However, during the 2019 outbreak, the incidence peak was observed in August when more than 50% of all cases were reported in this month. The peak of dengue incidence appears to have shifted from August to September compared to the last decade (until 2010) [17]. Interestingly, in the current (2022) outbreak, case reporting peaked in October. A recent study has projected that dengue transmission could be extended all year round at the end of 21 century under the consistently changing climate of Bangladesh [45].

### Future disease risks of dengue in Bangladesh

#### Expansion of dengue to non-endemic regions

**Fig. 3:**
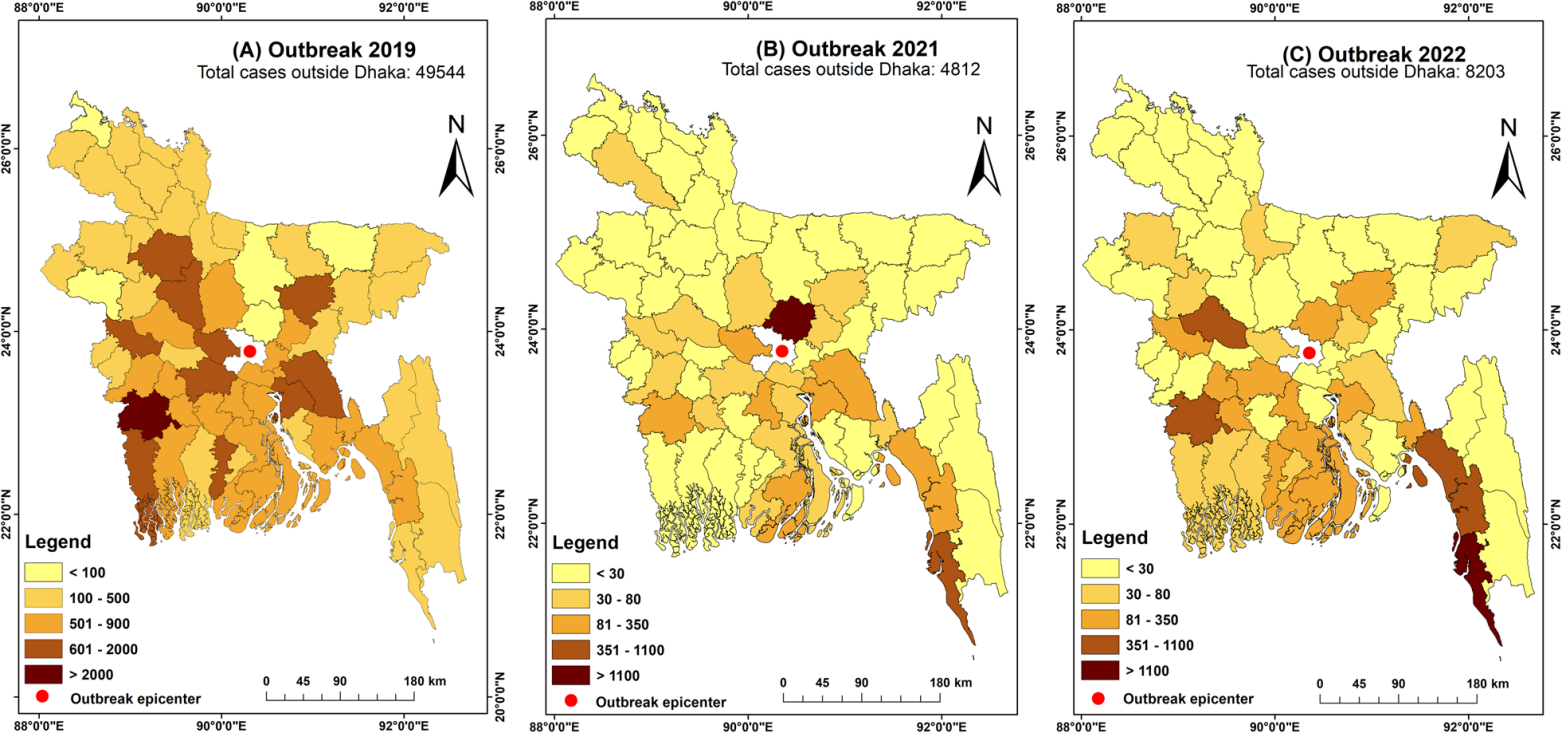
Maps showing the districtwise spatial distribution of dengue reported cases in 2019 (**A**), 2021 (**B**) & 2022 (**C**) outbreaks in Bangladesh excluding cases from historical outbreak epicenter Dhaka city and emerging epicenter Rohingya Refugee camps. Publicly available data were obtained from the Directorate general of health services, Bangladesh

The evidence of dengue spreading in non-endemic regions is mounting due to three consecutive outbreaks in 2019, 2021, and 2022. During the largest 2019 outbreak, nearly half of all cases (48.4%) were reported from all 64 districts of Bangladesh. (Fig. 3A). Similar circumstances were seen in the outbreak of 2021, where 20.4% of cases were recorded from locations outside Dhaka [10]. Nevertheless, the true magnitude of the 2021 outbreak (28,429 cases and 105 deaths) remains unknown as it was masked by the COVID-19 pandemic (Fig. 3B) [14]. In the 2022 outbreak, over one-third (37.5%) of all cases were reported from outside of Dhaka city (Fig. 3C).

*Evidence from the outbreak in Rohingya camps* For the first time, apart from Dhaka city, a massive outbreak is occurring in Rohingya refugee camps, which provide shelters to nearly 1.2 million forcibly displaced Myanmar nationals in a highly restricted area of Ukhiya, Cox’s Bazar district [46]. The first case of dengue in Rohingya camps was detected in 2017, which was followed by a small outbreak in 2021 (1530 cases and three deaths) [46]. However, the 2022 outbreak occurred on an even bigger scale, with 13,886 Rohingya people being infected and 27 deaths recorded [47]. Highly crowded Rohingya camps with a poor sanitation system may provide an optimal breeding ground for mosquitoes, and therefore, people are at higher risk of severe dengue because of secondary infection in upcoming years. Besides, it also appears that dengue is locally transmitted among Bangladeshi citizens at Cox’s Bazar since nearly 42.7% (1466/3427 cases) of all regional cases (Chittagong division) are recorded in this district [10].

The geographic spread of dengue is determined by a complex interaction of environmental and climatic factors, population density, and the abundance and adaptation of mosquito vector species and viruses [48]. The survival and proliferation of *Aedes* sp. mosquitoes are highly temperature-sensitive, with an optimum temperature range between 23 °C and 29 °C [49]. Importantly, all regions of Bangladesh are climatically almost similar, with an average mean temperature ranging from 22 °C to 33 °C during summer (March to October), making it ideal for the spread of dengue [50]. Accordingly, a survey of dengue vectors conducted in 2014–2015 found that *Aedes aegypti* was more prevalent in urban settings, whereas *Aedes albopictus* was more prevalent in rural Bangladesh [12].

Based on the data of recent outbreaks, some non-endemic southern districts (e.g., Jessore, Pabna, and Cox’s Bazar) appear to be potential dengue hotspots in terms of the frequency of cases reported (Fig. 3). Whether these district-level cases were either locally transmitted or had a travel history to Dhaka is mainly unknown. It is essential to consider that travel history from the Dhaka megacity to peripheral districts may substantially contribute to disseminating virus-carrying mosquitoes to other regions. In the last decade, major outbreaks (dengue, chikungunya, and COVID-19) coincided with annual mass migration during religious festivals (Eid) when half of the population (10–12 million) left Dhaka city [51]. Our prior study, for instance, showed that as compared to the rest of the country, the incidence of dengue cases declined drastically (nearly 24-fold) in Dhaka during the 2019 outbreak after 2 weeks of Eid exodus, while it significantly increased (four- to seven-folds) in some southern districts [15].

**Fig. 4:**
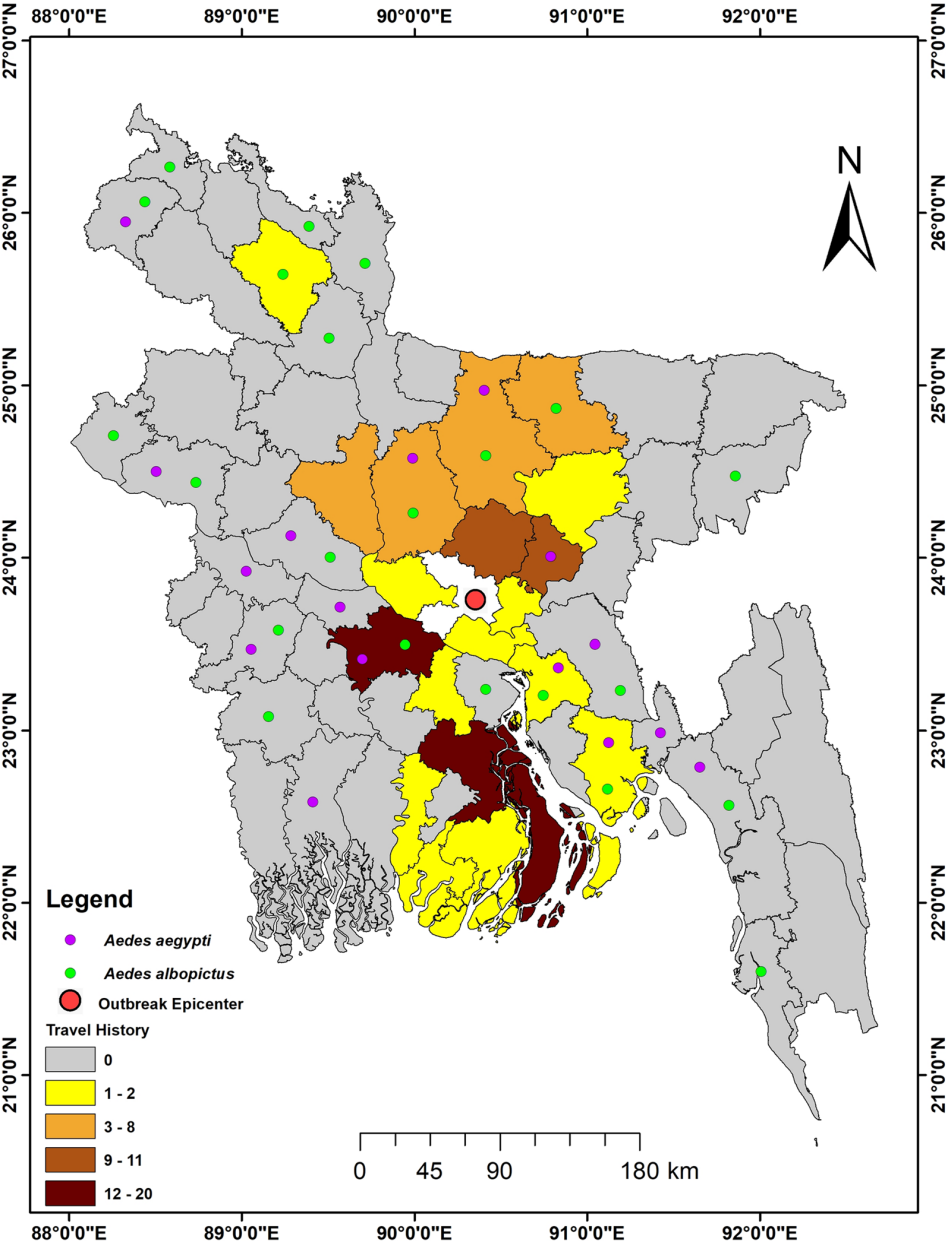
Map of Bangladesh showing dengue reported cases without travel history away from epicenter Dhaka in the 2019 outbreak along with species distribution of *Ae. aegypti* and *Ae. Albopictus*. Secondary data for dengue cases [10] and mosquitos were retrieved from the previous studies [11], respectively

Interestingly, this higher incidence pattern from outside Dhaka was sustained until the end of the outbreak, suggesting local dengue transmission. In line with this observation, based on our multi-centered hospital-based study, we found 262 dengue cases (district-level) admitted to hospitals in Dhaka without a recent travel history (Fig. 4) [23]. Another recent finding confirmed local dengue transmission in a northern district during the 2019 outbreak [39]. All these evidences indicate that dengue is spreading to non-endemic regions in Bangladesh.

It is important to note that Bangladesh is undergoing rapid and unplanned urbanization in all peripheral district towns [52]. For instance, we found that one least developed district town has urbanized by over 600% in two decades [53]. Urbanization is considered an essential driver for mosquito-borne disease transmission, since it creates mosquito breeding habitats, increases the chance of vector–human interaction, and promotes spatial distribution [54]. Taking all the factors into account, the risk of dengue outbreaks is impending across Bangladesh.

### Is Bangladesh prepared to tackle large-scale dengue outbreaks in non-endemic regions?

The resource-limited healthcare system of the country is expected to be profoundly impacted by the spread of dengue infection to non-endemic areas. Notably, in Bangladesh, the per capita health expenditure stands at US$110 [55], whereas the average per capita health expenditure in South Asia amounts to US$401. Since the massive outbreak in 2019, Bangladesh health authority has taken some initiatives, including training on case management of healthcare professionals, availability of rapid detection kits (NS1-based) and allocation of some beds for dengue patients at district-level public hospitals, and revision of dengue clinical management guidelines [56]. However, a study revealed that 78% of all hospitalized patients in previous outbreaks (2000–2017) sought healthcare support at private hospitals, and the case fatality was four times lower than in public hospitals [57]. This is consistent with the report by the World Bank, which showed that nearly 70% of patients seek medical services in the private healthcare sector in Bangladesh [58]. Supportive treatment costs are much higher in private hospitals, which are not generally affordable to people in Bangladesh.

Severe dengue patients require good supportive care, while more serious patients require intensive care unit (ICU) assistance. The tertiary healthcare system necessary for treating severe dengue cases is mostly centered in major cities, particularly Dhaka [14]. Along with poor accessibility of ICU facilities at the district level, there are also resource constrain for serological lab tests/lab facilities. Therefore, a district-level healthcare facility is not well-equipped to tackle severe dengue outbreaks in most non-endemic regions of Bangladesh. As a backdrop, the existing poor healthcare system could be collapsed if dengue outbreaks are widespread across the country or coincide with other viral disease outbreaks, such as the COVID-19 pandemic [14].

Even though Dhaka city is hyperendemic to dengue, no coordinated vector control policy has yet been devised. The city corporation lacks the necessary resources (infrastructure and workforce), and community engagement is also inadequate for dengue prevention [59]. A recent survey has shown that most people have heard about dengue but are unaware of mosquito breeding sites and their biting habits [19].

## Limitations

In this study, there are some limitations. Firstly, since this study did not follow a systematic review methodology, the search strategy was not comprehensive. Secondly, due to lack of publications in indexed journals, we had to consider local journals which were peer-reviewed but were not indexed in major databases (Pubmed, Scopus).

## Conclusion and future directions

Since the first recorded outbreak in 2000, dengue epidemiology has shown the classic epidemic pattern with more frequent and larger outbreaks and progressive geographic expansion. A periodic shift in the circulating serotypes of DENV was observed in the last two decades. Recent major outbreaks are found to be associated with the emergence of serotype DENV-3, which has been unnoticed for a long time. Consequently, changes in serotypes might be attributed to increased severity in clinical manifestation in recent years. Given the rapid and unplanned urbanization, the presence of the *Aedes* mosquito, and the climatic suitability of vector adaptation and DENV transmission, the expansion of dengue outbreaks in different geographic regions in Bangladesh is imminent. In the context of Bangladesh, our recommendations are as follows:

1. *Strengthening and extension of the surveillance system* The inherently weak existing surveillance system and risk management activities are inadequate to deal with impending dengue risks in Bangladesh, with a flawed healthcare system. Laboratory-based active surveillance is required to supplement the current hospital-based passive surveillance system. The surveillance system should include all hospitals/clinics and diagnostic labs in Dhaka city and at the district level. Low-cost laboratory facilities should be included in the surveillance system to make it affordable to the community.
2. *Molecular early warning system for predicting a major outbreak* Even though *Aedes* mosquitos play a critical role in DENV transmission, vector indices (e.g. density of mosquito population) correlate poorly for predicting outbreaks [60, 61]. Previous studies have revealed that the circulation of dengue serotypes in the environment typically follows a cyclic pattern, with distinct serotypes occasionally re-emerging to dominate and the introduction of a new serotype or replacement of an existing serotype or clade is associated with the occurrence of outbreaks [62]. Moreover, there is mounting evidence that natural vertical transmission (female mosquitoes to offspring) may serve as a maintenance mechanism for DENV during unfavorable conditions (i.e., silent circulation of a serotype in the absence of an outbreak) and implicate in an outbreak. Therefore, molecular tracking of DENV in mosquitoes and human patients during the outbreak and interepidemic seasons could be helpful as an early warning system for outbreaks.
3. *Capacity building at the community level* Empowering the community is one of the most critical components of the dengue prevention strategy. Social and ecological factors should be considered to develop a sustainable dengue mitigation strategy. Emphasis should be given to develop community capacity, including identifying risks and undertaking interventions (such as integrated vector control and strengthening the local health system). The government alone cannot implement the dengue mitigation strategy effectively; thus, public–private partnerships can play a pivotal role in community-level capacity building. In this context, cost-effective school- and mosque-based (since Bangladesh is a conservative Muslim society where mosques play an essential role in community engagement) citizen science initiatives could be cost-effective for raising awareness on dengue and *Aedes* mosquito surveillance at the community level [63, 64]. The involvement of social media and the use of electronic media like television could be a useful resource to enhance health awareness and health education among community people.
4. *Socio-cultural determinants* Large population-based studies are warranted to understand community perceptions, health-seeking behaviors, and disease severity and identify gaps and the vulnerable population at risk.

In nutshell, we have depicted the overall dengue situation of Bangladesh and identified gaps in dengue management, and provided future perspectives to prevent impending outbreaks across. Our findings would be helpful for other similar settings elsewhere in the world.

## Supplementary Information

**Additional file 1. File 1:** One database search strategy/results. **File 2:** The list of excluded and included studies. **File 3:** SANRA assessment score.

## Data Availability

Data included in the manuscript.
