# Supplementary material for: Twenty-two years of dengue outbreaks in Bangladesh: epidemiology, clinical spectrum, serotypes, and future disease risks
Source: Trop Med Health. 2023 Jul 11;51:37. doi: 10.1186/s41182-023-00528-6 (PMC10334535; doi:10.1186/s41182-023-00528-6)
Supplement: Supplementary file 1 — Additional file 1. File 1: One database search strategy/results. File 2: The list of excluded and included studies. File 3: SANRA assessment score. [file 41182_2023_528_MOESM1_ESM.docx]

**Supplementary file 1: One database search strategy/results**

**Pubmed search details**

**Date searched: 01/01/2023**

((Dengue) AND (Bangladesh)) AND (("2000/01/01"[Date - Publication] : "2022/12/30"[Date - Publication]))

Total hit: 260

Link: https://pubmed.ncbi.nlm.nih.gov/?term=%28%28Dengue%29+AND+%28Bangladesh%29%29+AND+%28%28%222000%2F01%2F01%22%5BDate+-+Publication%5D+%3A+%222022%2F12%2F30%22%5BDate+-+Publication%5D%29%29&sort=pubdate

**Supplementary file 2:** The list of excluded and included studies

**Studies of potential interest (n = 85), Excluded studies = (n=60)**

| Title | Author | Publication date |
| --- | --- | --- |
| A Bayesian approach for estimating under-reported dengue incidence with a focus on non-linear associations between climate and dengue in Dhaka, Bangladesh | Sharmin S., Glass K., Viennet E., Harley D. | 2018 |
| A retrospective study on the socio-demographic factors and clinical parameters of dengue disease and their effects on the clinical course and recovery of the patients in a tertiary care hospital of Bangladesh | Prattay K.M.R., Sarkar M.R., Shafiullah A.Z.M., Islam M.S., Raihan S.Z., Sharmin N. | 2022 |
| An Association between Rainy Days with Clinical Dengue Fever in Dhaka, Bangladesh: Findings from a Hospital Based Study | Rahman KM | 2020 |
| Analysis and identification of genomic and immunogenic features of dengue serotype 3 genomes obtained during the 2019 outbreak in Bangladesh | Shirin T., Akram A., Hasan S., Rahman A., Sultana S., Alam A.N., Khan M.H., Hossain S., Flora M.S., Hossain M. | 2022 |
| Climate change and dengue fever knowledge, attitudes and practices in Bangladesh: A social media-based cross-sectional survey | Siddikur Rahman M., Karamehic-Muratovic A., Baghbanzadeh M., Amrin M., Zafar S., Rahman N.N., Shirina S.U., Haque U. | 2021 |
| Climate variability, dengue vector abundance and dengue fever cases in dhaka, bangladesh: A time-series study | Islam S., Emdad Haque C., Hossain S., Hanesiak J. | 2021 |
| Climatic factors influencing dengue cases in Dhaka city: A model for dengue prediction | Karim M.N., Munshi S.U., Anwar N., Alam M.S. | 2012 |
| Clinical and hematological profiles of children with dengue residing in a non-endemic zone of Bangladesh | Islam S | 2022 |
| Clinical and laboratory observations associated with the 2000 dengue outbreak in Dhaka, Bangladesh | Pervin M., Tabassum S., Ali M., Mamun K.Z., Islam N. | 2004 |
| Clinical Profile and Lab Findings of Dengue Fever in Children Admitted in a Tertiary Care Hospital | Mutanabbi M | 2022 |
| Clinical profile and outcome of patients with dengue syndrome in hospital care | Mohammad H., Sarkar D.N., Amin M.R., Basher A., Ahmed T. | 2011 |
| Clinical profile, haematological changes and outcomes of dengue patients in dengue outbreak-2019 in Jashore, Bangladesh - An observational study | Ali M., Acherjya G.K., Islam A.K.M.M., Alam A.B.M.S., Rahman S.M.S., Chowdhury R.S., Shamsuzzaman M., Chakrabortty R., Roy G.C. | 2021 |
| Clinico-pathological profile of dengue syndrome: an experience in a tertiary care hospital, Dhaka, Bangladesh | Uddin M.N., Hossain M.M., Dastider R., Hasan Z., Ahmed Z., Dhar D.K. | 2014 |
| Community perspectives on dengue transmission in the City of Dhaka, Bangladesh | Dhar-Chowdhury P., Emdad Haque C., Michelle Driedger S., Hossain S. | 2014 |
| Correlates of Climate Variability and Dengue Fever in Two Metropolitan Cities in Bangladesh | Zahirul Islam M | 2018 |
| COVID-19 and Dengue Co-epidemic During the Second Wave of the Pandemic in Bangladesh: A Double Blow for an Overburdened Health-Care System | Patwary MM | 2022 |
| COVID-19 and mosquito-borne diseases in Bangladesh: Is the pandemic masking another sinister public health threat? | Mou S.I., Kabir H., Hasan M.K. | 2022 |
| COVID-19 pandemic, dengue epidemic, and climate change vulnerability in Bangladesh: Scenario assessment for strategic management and policy implications | Rahman M.M., Bodrud-Doza M., Shammi M., Md Towfiqul Islam A.R., Moniruzzaman Khan A.S. | 2021 |
| Dengue and dengue haemorrhagic fever in children during the 2000 outbreak in Chittagong, Bangladesh | Ahmed F.U., Mahmood C.B., Sharma J.D., Hoque S.M., Zaman R., Hasan M.S. | 2001 |
| Dengue Fever Outbreak in a Recreation Club, Dhaka, Bangladesh | Wagatsuma Y., Breiman R.F., Hossain A., Rahman M. | 2004 |
| Dengue Fever Responses in Dhaka City, Bangladesh: A Cross-Sectional Survey | Rahman M.M., Islam A.R.M.T., Khan S.J., Tanni K.N., Roy T., Islam M.R., Rumi M.A.A.R., Sakib M.S., Abdul Quader M., Bhuiyan N.-U.-I., Chisty M.A., Rahman F., Alam E. | 2022 |
| Dengue in Dhaka, Bangladesh: Hospital-based cross-sectional KAP assessment at Dhaka North and Dhaka South City Corporation area | Abir T., Ekwudu O., Kalimullah N.A., Nur-A Yazdani D.M., Mamun A.A., Basak P., Osuagwu U.L., Yukthamarani Permarupan P., Milton A.H., Talukder S.H., Agho K.E. | 2021 |
| Dengue infection in Dhaka City, Bangladesh. | Sharmin R., Tabassum S., Mamun K.Z., Nessa A., Jahan M. | 2013 |
| Dengue outbreak 2019: clinical and laboratory profiles of dengue virus infection in Dhaka city | Mahmood R., Benzadid M.S., Weston S., Hossain A., Ahmed T., Mitra D.K., Ahmed S. | 2021 |
| Dengue outbreaks in Bangladesh: Historic epidemic patterns suggest earlier mosquito control intervention in the transmission season could reduce the monthly growth factor and extent of epidemics | Haider N, Chang YM, Rahman M, Zumla A, Kock RA | 2021 |
| Detection of dengue epidemic in Dhaka, Bangladesh by a neuro fuzzy approach | Arifuzzaman M., Shaon M.F.I., Islam M.J., Rahman R.M. | 2016 |
| Devastating dengue outbreak amidst COVID-19 pandemic in Bangladesh: an alarming situation | Hasan M.M., Sahito A.M., Muzzamil M., Mohanan P., Islam Z., Billah M.M., Islam M.J., Essar M.Y. | 2022 |
| Diversity of Dengue Virus Serotypes in Dhaka City: From 2017 to 2021 | Rahim R, Hasan A, Hasan N, Nakayama EE, Shioda T, Rahman M | 2021 |
| Emerging and re-emerging infectious diseases: Bangladesh perspective | Kahhar M.A. | 2012 |
| Epidemiological factors of dengue hemorrhagic fever in Bangladesh | Rasul C.H., Ahasan H.A.M.N., Rasid A.K.M.M., Khan M.R.H. | 2002 |
| Genome sequence of a dengue virus serotype 2 strain identified during the 2019 outbreak in bangladesh | Malaker R., Sajib M.S.I., Malaker A.R., Rahman H., Hooda Y., Hasanuzzaman M., Saha S.K., Saha S. | 2021 |
| Genotype replacement of dengue virus type 3 and clade replacement of dengue virus type 2 genotype Cosmopolitan in Dhaka, Bangladesh in 2017 | Suzuki K., Phadungsombat J., Nakayama E.E., Saito A., Egawa A., Sato T., Rahim R., Hasan A., Lin M.Y.-C., Takasaki T., Rahman M., Shioda T. | 2019 |
| Geostatistical mapping of the seasonal spread of under-reported dengue cases in Bangladesh | Sharmin S., Glass K., Viennet E., Harley D. | 2018 |
| High prevalence and genetic diversity of Plasmodium malariae and no evidence of Plasmodium knowlesi in Bangladesh | Fuehrer H.-P., Swoboda P., Harl J., Starzengruber P., Habler V.E., Bloeschl I., Haque R., Matt J., Khan W.A., Noedl H. | 2014 |
| Hospital Seroprevalence of Dengue Virus Infection among Adults of Urban Dhaka | Hoque M.M., Robed Amin Md., Biswas S., Islam M.R. | 2021 |
| Hospital-based prevalence of malaria and dengue in febrile patients in Bangladesh | Faruque L.I., Zaman R.U., Alamgir A.S.M., Gurley E.S., Haque R., Rahman M., Luby S.P. | 2012 |
| How dengue vector Aedes albopictus (Diptera: Culicidae) survive during the dry season in Dhaka City, Bangladesh? | Chowdhury R., Chowdhury V., Faria S., Huda M.M., Laila R., Dhar I., Maheswary N.P., Dash A.P. | 2014 |
| Hydroclimatological variability and dengue transmission in Dhaka, Bangladesh: A time-series study | Hashizume M., Dewan A.M., Sunahara T., Rahman M.Z., Yamamoto T. | 2012 |
| Insecticide resistance status of Aedes aegypti in Bangladesh | Al-Amin H.M., Johora F.T., Irish S.R., Hossainey M.R.H., Vizcaino L., Paul K.K., Khan W.A., Haque R., Alam M.S., Lenhart A. | 2020 |
| Interaction of mean temperature and daily fluctuation influences dengue incidence in Dhaka, Bangladesh | Sharmin S., Glass K., Viennet E., Harley D. | 2015 |
| Is the COVID-19 pandemic masking dengue epidemic in Bangladesh? | Rahman M.T., Sobur M.A., Islam M.S., Toniolo A., Nazir K.H.M.N.H. | 2020 |
| Knowledge and beliefs of the city dwellers regarding dengue transmission and their relationship with prevention practices in Dhaka city, Bangladesh | Bashar K | 2020 |
| Knowledge attitude and practice on dengue among households in rural areas [north dharmapur, gaibandha bangladesh] | Rahman M.M., Farhana M.M., Majumder S., Akter F., Rajib M.A.H., Haque M.A., Afroz T. | 2020 |
| Knowledge, attitude and prevention practices of garment factory workers regarding the largest Dengue outbreak on record in Bangladesh | Das S., Rahman M.M., Rahaman M.M., Noor M., Akter M., Uddin M.J., Alam K.J., Rahman M.M., Hossain F.M.A., Islam M.T., Ashour H.M. | 2022 |
| Knowledge, Attitude, and Practices towards Dengue Fever among University Students of Dhaka City, Bangladesh | Rahman M.M., Khan S.J., Tanni K.N., Roy T., Chisty M.A., Islam M.R., Rumi M.A.A.R., Sakib M.S., Quader M.A., Bhuiyan M.N.-U.-I., Rahman F., Alam E., Islam A.R.M.T. | 2022 |
| Knowledge, Attitudes, and Practices in Relation to Mosquito?Borne Diseases in Bangladesh | Mobin M., Khan M., Anjum H., Rahman H., Marzan M., Islam M.A. | 2022 |
| Molecular and serological study of dengue virus-infected patients attending a tertiary hospital of Dhaka city, Bangladesh (2013 to 2016) | Akther T., Muraduzzaman A.K.M., Parvin S., Tabssum S., Munshi S. | 2019 |
| Origin of dengue type 3 viruses associated with the dengue outbreak in Dhaka, Bangladesh, in 2000 and 2001 | Podder G., Breiman R.F., Azim T., Thu H.M., Velathanthiri N., Mai L.Q., Lowry K., Aaskov J.G. | 2006 |
| Outbreak of Dengue Amid the COVID-19 Pandemic: An Emerged Crisis for Bangladesh | Islam M.T., Rahman M., Sadia F.J. | 2022 |
| Possible drivers of the 2019 dengue outbreak in Bangladesh: The need for a robust community-level surveillance system | Ahsan A., Haider N., Kock R., Benfield C. | 2021 |
| Prediction of dengue annual incidence using seasonal climate variability in Bangladesh between 2000 and 2018 | Hossain MP | 2022 |
| Predominance of the DEN-3 genotype during the recent dengue outbreak in Bangladesh | Aziz MM | 2002 |
| Reemergence of dengue virus in Bangladesh: Current fatality and the required knowledge | Noor R. | 2020 |
| Seasonal distribution and climatic correlates of dengue disease in Dhaka, Bangladesh | Morales I., Salje H., Saha S., Gurley E.S. | 2016 |
| Sero-epidemiological study of dengue/dengue haemorrhagic fever in a metropolitan hospital in Bangladesh | Bin Yunus E., Banu D., Kanak Talukder K.R., Hossain Chowdhury M.J., Mannan Bangali A., Montanari R.M. | 2002 |
| Serologic Evidence of Dengue Infection before Onset of Epidemic, Bangladesh | Hossain M.A., Khatun M., Arjumand F., Nisaluk A., Breiman R.F. | 2003 |
| Serological evidence of dengue fever in the Bangladesh Republic | Gaidamovich S.Y., Siddiqi S.M., Haq F., Klisenko G.A., Melnikova E.E., Obukhova V.R. | 1980 |
| Seropositivity and pattern of dengue infection in Dhaka city | Rahman MT | 2007 |
| Seroprevalence of dengu infections amongst the children. | Chowdhury A.Q., Miah R.A., Akhtar N., Jubayer S.M. | 2004 |
| Sociodemographic and Clinico-laboratory Profile of Expanded Dengue Syndrome: Experience from a Tertiary Hospital of Dhaka, Bangladesh | Salma U | 2021 |

**Included studies = (n=25)**

| Circulating dengue virus serotypes in Bangladesh from 2013 to 2016 | Muraduzzaman A.K.M., Alam A.N., Sultana S., Siddiqua M., Khan M.H., Akram A., Haque F., Flora M.S., Shirin T. | 2018 |
| --- | --- | --- |
| Clinical profile and outcome of dengue hemorrhagic fever in a Tertiary Care Hospital in Dhaka | Arif K.M., Mohammed F.R., Nur Z., Shams Md.Z., Alam Md.B., Uddin Md.J., Nazmul Ahasan H.A.M. | 2009 |
| Clinical Profile of Dengue Fever in Children | Alam AS, Sadat SA, Swapan Z, Ahmed AU, Karim MN, Paul H, et al. | 2009 |
| Clinical spectrum and predictors of severity of dengue among children in 2019 outbreak: a multicenter hospital-based study in Bangladesh | Khan M.A.S., Al Mosabbir A., Raheem E., Ahmed A., Rouf R.R., Hasan M., Alam F.B., Hannan N., Yesmin S., Amin R., Ahsan N., Anwar S., Afroza S., Hossain M.S. | 2021 |
| Clinico-epidemiologic characteristics of the 2019 dengue outbreak in Bangladesh | Hasan MJ | 2021 |
| Co-circulation of dengue virus type 3-genotype I and type 2-Cosmopolitan genotype in 2018 outbreak in Dhaka, Bangladesh | Ahmad F.U., Paul S.K., Aung M.S., Mazid R., Alam M., Ahmed S., Haque N., Hossain M.A., Paul S., Sharmin R., Kobayashi N. | 2020 |
| Comparison of clinical manifestation of dengue fever in Bangladesh: an observation over a decade | Hasan M.J., Tabassum T., Sharif M., Khan M.A.S., Bipasha A.R., Basher A., Islam M.R., Amin M.R. | 2021 |
| COVID-19 onslaught is masking the 2021 dengue outbreak in Dhaka, Bangladesh | Hossain M.S., Amin R., Al Mosabbir A. | 2022 |
| Dengue epidemic in a non-endemic zone of Bangladesh: Clinical and laboratory profiles of patients | Rafi A., Mousumi A.N., Ahmed R., Chowdhury R.H., Wadood A., Hossain G. | 2020 |
| Dengue in a crowded megacity: Lessons learnt from 2019 outbreak in Dhaka, Bangladesh | Hossain MS | 2020 |
| Dengue outbreak 2000 in Bangladesh: From speculation to reality and exercises | Yunus E.B, Mahmood M.A.H., Rahman M.M., Chowdhury A.R., Talukder K.R. | 2001 |
| Dengue prevention and control: Bangladesh context. | Rahman SMM, Hossain SM, Jahan MUZ | 2019 |
| Dengue seroprevalence, seroconversion and risk factors in Dhaka, Bangladesh | Dhar-Chowdhury P., Paul K.K., Haque C.E., Hossain S., Lindsay L.R., Dibernardo A., Brooks W.A., Drebot M.A. | 2017 |
| Dengue Situation in Bangladesh: An Epidemiological Shift in terms of Morbidity and Mortality | Mutsuddy P., Tahmina Jhora S., Shamsuzzaman A.K.M., Kaisar S.M.G., Khan M.N.A., Dhiman S. | 2019 |
| Dengue transmission risk in a changing climate: Bangladesh is likely to experience a longer dengue fever season in the future | Paul K.K., Macadam I., Green D., Regan D.G., Gray R.T. | 2021 |
| First outbreak of dengue hemorrhagic fever, Bangladesh | Rahman M., Rahman K., Siddque A.K., Shoma S., Kamal A.H.M., Ali K.S., Nisaluk A., Breiman R.F. | 2002 |
| Frequency of dengue infection in febrile patients attended Dhaka Medical College Hospital during January to December, 2018 | Sultana N, Fatema N, Hossain MZ, Rahman MA, Nehar N, Yeasmin MM, et al | 2018 |
| Isolation and serotyping of dengue viruses by mosquito inoculation and cell culture technique: An experience in Bangladesh | Pervin M., Tabassum S., Sil B.K., Islam Md.N. | 2003 |
| Knowledge, awareness and preventive practices of dengue outbreak in Bangladesh: A countrywide study | Hossain M.I., Alam N.E., Akter S., Suriea U., Aktar S., Shifat S.K., Islam M.M., Aziz I., Islam M.M., Islam M.S., Mohiuddin A.K.M. | 2021 |
| Largest dengue outbreak of the decade with high fatality may be due to reemergence of DEN-3 serotype in Dhaka, Bangladesh, necessitating immediate public health attention | Shirin T., Muraduzzaman A.K.M., Alam A.N., Sultana S., Siddiqua M., Khan M.H., Akram A., Sharif A.R., Hossain S., Flora M.S. | 2019 |
| Megacity-centric mass mobility during Eid holidays: a unique concern for infectious disease transmission in Bangladesh | Hossain M.S. | 2022 |
| Molecular characterization and clinical evaluation of dengue outbreak in 2002 in Bangladesh | Islam M.A., Ahmed M.U., Begum N., Chowdhury N.A., Khan A.H., Parquet M. del C., Bipolo S., Inoue S., Hasebe F., Suzuki Y., Morita K. | 2006 |
| Nationally-representative serostudy of dengue in Bangladesh allows generalizable disease burden estimates | Salje H., Paul K.K., Paul R., Rodriguez-Barraquer I., Rahman Z., Alam M.S., Rahman M., Al-Amin H.M., Heffelfinger J., Gurley E. | 2019 |
| Nationwide distribution of dengue virus type 3 (Denv-3) genotype i and emergence of denv-3 genotype iii during the 2019 outbreak in bangladesh | Titir S.R., Paul S.K., Ahmed S., Haque N., Nasreen S.A., Hossain K.S., Ahmad F.U., Nila S.S., Khanam J., Nowsher N., Amin A.M.M.A., Khan A.U., Aung M.S., Kobayashi N. | 2021 |
| Sera-epidemiology of Dengue Virus Infection in Clinically Suspected Patients Attended in Dhaka Medical College Hospital During January to December 2016 | Pervin M, Sweety AA, Hossain MZ, Sharmin R, Fatema N, Rahman MA, et al. | 2016 |
| The emergence of dengue in Bangladesh: epidemiology, challenges and future disease risk | Sharmin S | 2015 |

**Supplementary file 3:** SANRA assessment score

Scale for the Assessment of Narrative Review Articles – SANRA

Please rate the quality of the narrative review article in question, using categories 0–2 on the following scale. For each aspect of   quality, please choose the option which best fits your evaluation, using categories 0 and 2 freely to imply general low and high quality.  These are not intended to imply the worst or best imaginable quality.

**1) Justification of the article’s importance for the readership**

  The importance is not justified. 0

2

The importance is alluded to, but not explicitly justified. 1

The importance is explicitly justified. 2

**2) Statement of concrete aims or formulation of questions**

No aims or questions are formulated. 0

2

Aims are formulated generally but not concretely or in terms of clear questions. 1

One or more concrete aims or questions are formulated. 2

**3) Description of the literature search**

 The search strategy is not presented. 0

1

The literature search is described briefly. 1

The literature search is described in detail, including search terms and inclusion criteria. 2

**4) Referencing**

  Key statements are not supported by references. 0

2

The referencing of key statements is inconsistent. 1

Key statements are supported by references. 2

**5) Scientific reasoning**

*(e.g., incorporation of appropriate evidence, such as RCTs in clinical medicine)*

  The article’s point is not based on appropriate arguments. 0

2

Appropriate evidence is introduced selectively. 1

Appropriate evidence is generally present. 2

**6) Appropriate presentation of data**

*(e.g., absolute vs relative risk; effect sizes without confidence intervals)*

  Data are presented inadequately. 0

2

Data are often not presented in the most appropriate way. 1

Relevant outcome data are generally presented appropriately. 2

11

**Sum score**

SANRA-explanations and instructions

This scale is intended to help editors assess the quality of a narrative review article based on formal criteria accessible to the reader.  It cannot cover other elements of editorial decision making such as degree of originality, topicality, conflicts of interest or the plausibility, correctness or completeness of the content itself. SANRA is an instrument for editors, authors, and reviewers evaluating individual manuscripts. It may also help editors to document average manuscript quality within their journal and researchers to document the   manuscript quality, for example in peer review research. Using only three scoring options, 0, 1 and 2, SANRA is intended to provide a swift and pragmatic sum score for quality, for everyday use with real manuscripts, in a field where established quality standards have  previously been lacking. It is not designed as an exact measurement of the quality of all theoretically possible manuscripts. For this reason, the extreme values (0 and 2) should be used relatively freely and not reserved only for perfect or hopeless articles.

We recommend that users test-rate a few manuscripts to familiarize themselves with the scale, before using it on the intended group of manuscripts. Ratings should assess the totality of a manuscript, including the abstract. The following comments clarify how each question is designed to be used.

**Item 1 – Justification of the article’s importance for the readership**

Justification of importance for the readership must be seen in the context of each journal’s readership.  Consider how well the manuscript outlines the clinical problem and highlights unanswered questions or evidence gaps – thoroughly (2), superficially (1), or not at all (0).

**Item 2 – Statement of concrete/specific aims or formulation of questions**

A good paper will propose one or more specific aims or questions which will be dealt with or topics which will be reviewed. Please rate whether this has been done thoroughly and clearly (2), vaguely or unclearly (1), or not at all (0).

**Item 3 – Description of the literature search**

A convincing narrative review will be transparent about the sources of information on which the text is based. Please rate the degree to which you think this has been achieved. To achieve a rating of 2, it is not necessary to describe the literature search in as much de tail as for a systematic review (searching multiple databases, including exact descriptions of search history, flowcharts, etc.), but it is necessary to specify search terms, and the types of literature included. A manuscript which only refers briefly to its literature search would score 1, while one not mentioning its methods would score 0.

**Item 4 – Referencing**

No manuscript references all statements. However, those that are essential for the arguments of the manuscript – “key statements” – should be backed by references in all or almost all cases. Exceptions could reasonably be made for rating purposes where a key statement has uncontroversial face-validity, such as “Diabetes is among the commonest causes of chronic morbidity worldwide.”  Please rate the completeness of referencing: for most or all relevant key statements (2), inconsistently (1), sporadically (0).

**Item 5 – Scientific reasoning**

The item describes the quality of the scientific point made. A convincing narrative review presents evidence for key arguments. It should mention study design (randomized controlled trial, qualitative study, etc), and where available, levels of evidence.   Please rate whether you feel this has been done thoroughly (2), superficially (1), or hardly at all (0). Unlike item 6, which is   concerned with the selection and presentation of concrete outcome data, this item relates to the use of evidence and of types of   evidence in the manuscript’s arguments.

**Item 6 – Appropriate presentation of data:**

This item describes the correct presentation of data central to the article’s argument. Which data are considered relevant varies from field to field. In some areas relevant data would be absolute rather than relative risks or clinical versus surrogate or intermediate end points. These outcomes must be presented correctly. For example, it is appropriate that effect sizes are accompanied by confidence intervals. Please rate how far the paper achieves this – thoroughgoingly (2), partially (1), or hardly at all (0). Unlike item 5, which relates to the use of evidence and of types of evidence in the manuscript’s arguments, this item is concerned with the selection and  presentation of concrete outcome data.

**Reference**

Baethge C, Goldbeck-Wood S, Mertens S: SANRA—a scale for the quality assessment of narrative review articles. Research Integrity and Peer Review (2019) 4:5 https://doi.org/10.1186/s41073-019-0064-8
